# Supplementary material for: Deterministic grayscale nanotopography to engineer mobilities in strained MoS2 FETs
Source: Nat Commun. 2024 Aug 13;15:6934. doi: 10.1038/s41467-024-51165-4 (PMC11322165; doi:10.1038/s41467-024-51165-4)
Supplement: Supplementary file 1 — Supplementary Information [file 41467_2024_51165_MOESM1_ESM.pdf]

# – Supplementary Information –

Deterministic grayscale nanotopography to engineer mobilities in strained MoS_2 FETs

Xia Liu[1,$,†,*], *Berke Erbas*[1,†], *Ana Conde-Rubio*[1,#], *Norma Rivano*[2,3], *Zhenyu Wang*[4], *Jin Jiang*[5], *Siiri Bienz*[6], *Naresh Kumar*[6], *Thibault Sohier*[7], *Marcos Penedo*[8], *Mitali Banerjee*[5], *Georg Fantner*[8], *Renato Zenobi*[6], *Nicola Marzari*[2,3,9], *Andras Kis*[4], *Giovanni Boero*[1], *Juergen Brugger*[1,*]

[1] Microsystems Laboratory, École Polytechnique Fédérale de Lausanne (EPFL), 1015 Lausanne, Switzerland

[2] Theory and Simulation of Materials (THEOS), École Polytechnique Fédérale de Lausanne (EPFL), 1015 Lausanne, Switzerland

[3] National Centre for Computational Design and Discovery of Novel Materials (MARVEL), École Polytechnique Fédérale de Lausanne (EPFL), 1015 Lausanne, Switzerland

[4] Laboratory of Nanoscale Electronics and Structures, École Polytechnique Fédérale de Lausanne (EPFL), 1015 Lausanne, Switzerland

[5] Laboratory of Quantum Physics, Topology and Correlations, École Polytechnique Fédérale de Lausanne (EPFL), 1015 Lausanne, Switzerland

[6] Department of Chemistry and Applied Biosciences, ETH Zurich, 8093 Zurich, Switzerland

[7] Laboratoire Charles Coulomb (L2C), Université de Montpellier, CNRS, Montpellier, France

[8] Laboratory for Bio- and Nano-Instrumentation, École Polytechnique Fédérale de Lausanne (EPFL), 1015 Lausanne, Switzerland

[9] Laboratory for Materials Simulations, Paul Scherrer Institute, 5232 Villigen PSI, Switzerland

[$] Current affiliation: School of Integrated Circuits and Electronics, MIIT Key Laboratory for Low-Dimensional Quantum Structure and Devices, Beijing Institute of Technology, Beijing 100081, China

[#] Current affiliation: Institute of Materials Science of Barcelona ICMAB-CSIC, Campus UAB, 08193 Bellaterra, Spain

[†] These authors (X.L. and B.E.) contribute equally.

* Corresponding authors: xia.liu@bit.edu.cn (X.L.), juergen.brugger@epfl.ch (J.B.)

Supplementary Note 1: Concept of tensile strain in patterned MoS_2

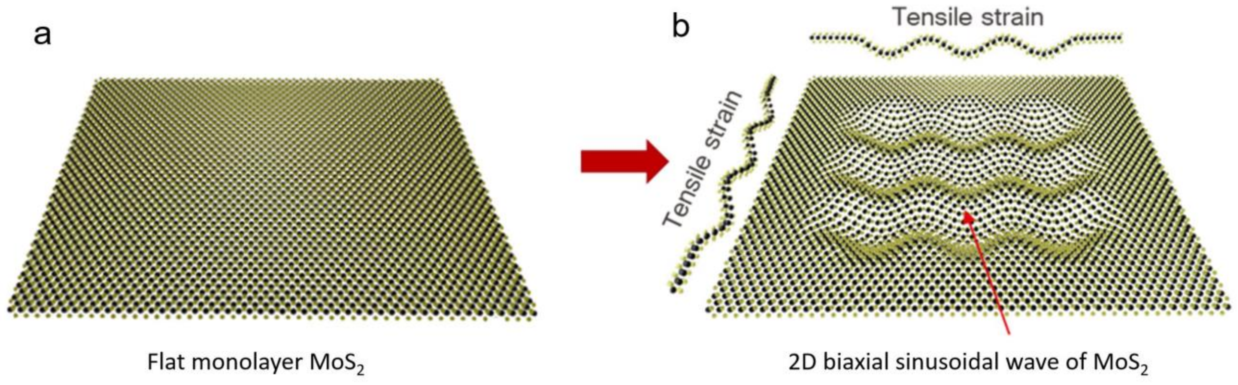

Supplementary Figure 1: Atomic structures of (a) a flat monolayer MoS_2 flake and (b) a MoS_2 flake with 2D biaxial sinusoidal wave pattern in the center, which introduces tensile strain.

Supplementary Note 2: Fabrication of grayscale 2D sinusoidal wave surface by t-SPL

T-SPL was used to locally sublimate a thermosensitive polymer, polyphthalaldehyde (PPA), controlling the depth of the patterns by adjusting the applied voltage (Supplementary Figure 2). In addition, a reader integrated into the cantilever provides simultaneous imaging of the patterned structures, and a feedback mechanism corrects the patterning depth for optimized grayscale lithography, enabling patterning with 10 nm lateral and 2 nm vertical resolution.

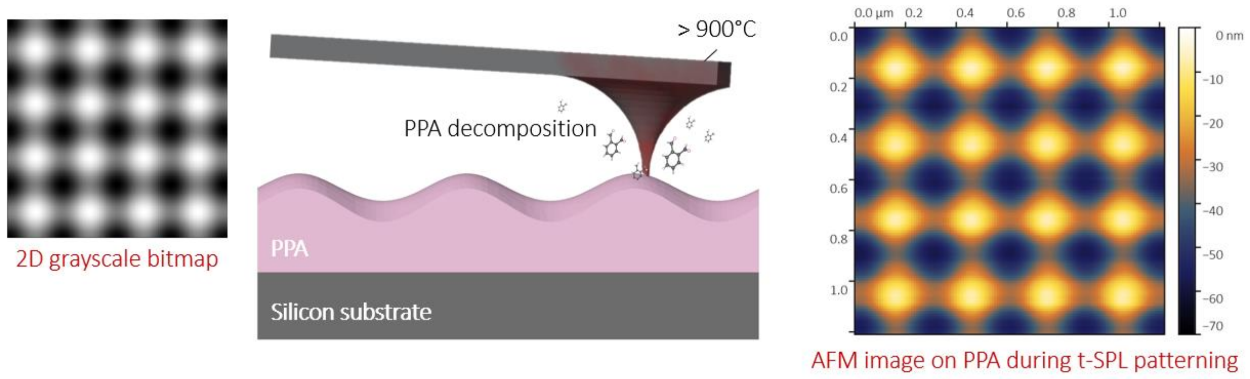

Supplementary Figure 2: Working principle of t-SPL-based nanopatterning on the thermosensitive resist. The heated nanotip locally removes a material (i.e. PPA) with the depth defined for each pixel on a 2D

grayscale bitmap (left). Since the thermal resist PPA directly sublimates upon exposure to heat without the need of any development step, the measurement of the cantilever deflection during t-SPL patterning provides simultaneous *in-situ* AFM imaging of the created patterns (right).

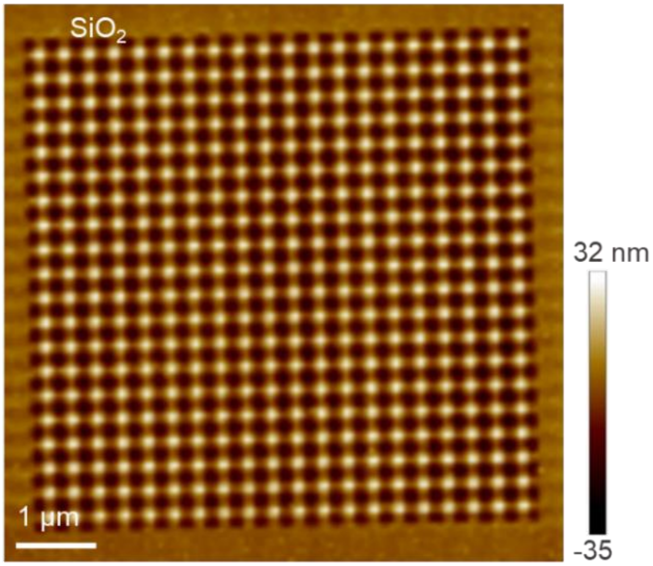

Supplementary Figure 3: AFM image of the biaxial sinusoidal SiO_2 substrate over an area of 6.5 μm × 6.5 μm.

Supplementary Note 3: Method of transferring MOCVD grown 2D flakes

Figure S5 shows the process flow of transferring as-grown monolayer MoS_2 flakes from the sapphire substrate. First, the substrate with flakes was spin-coated with PMMA (poly(methyl methacrylate)) A2 at a speed of 1500 rpm for 1 min and baked on a hot plate at 135℃ for 1 min for drying. A PDMS film (~1mm thick) was placed on top of PMMA. Then the edges of the PMMA film were scratched with a sharp tweezer. The sample was immersed in water for more than 20 min until the PDMS/PMMA/2D flakes stack detaches from the sapphire substrate. The mechanism of the detachment can be explained by the fact that water molecules can infiltrate into the interface between the hydrophilic sapphire surface and the PMMA surface so the 2D flakes coated by PMMA detach from the surface. Subsequently, another substrate is used to fish the stack in water and dry it at a moderate temperature of 50 ºC to avoid the expansion/detachment of the PDMS layer. In the end, the stack is immersed in acetone to dissolve PMMA (and make the PDMS

layer detach) overnight and annealed in high vacuum at 250 °C for 6 h to remove the PMMA residue. Thus, the flakes are transferred to the target substrate. The flake quality and transfer play an important part in device yield.

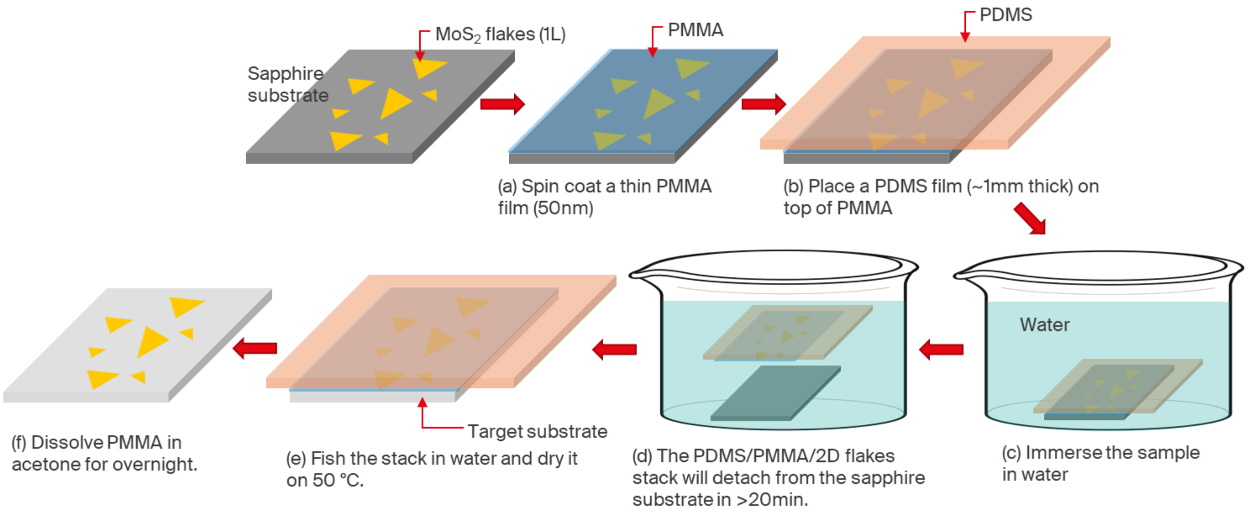

Supplementary Figure 4: Chip-level transfer of the flakes on sapphire chip to any other substrate. (a) Spin coating a thin layer of PMMA, (b) placing a PDMS film (~1 mm thick) on top of PMMA, (c) immersing the sample in water for >20 min, (d) detaching the PDMS/PMMA/2D flakes stack from the sapphire substrate, (e) fishing the stack in water using a target substrate and drying it at 50 ºC, and (f) immersing the stack in acetone for overnight.

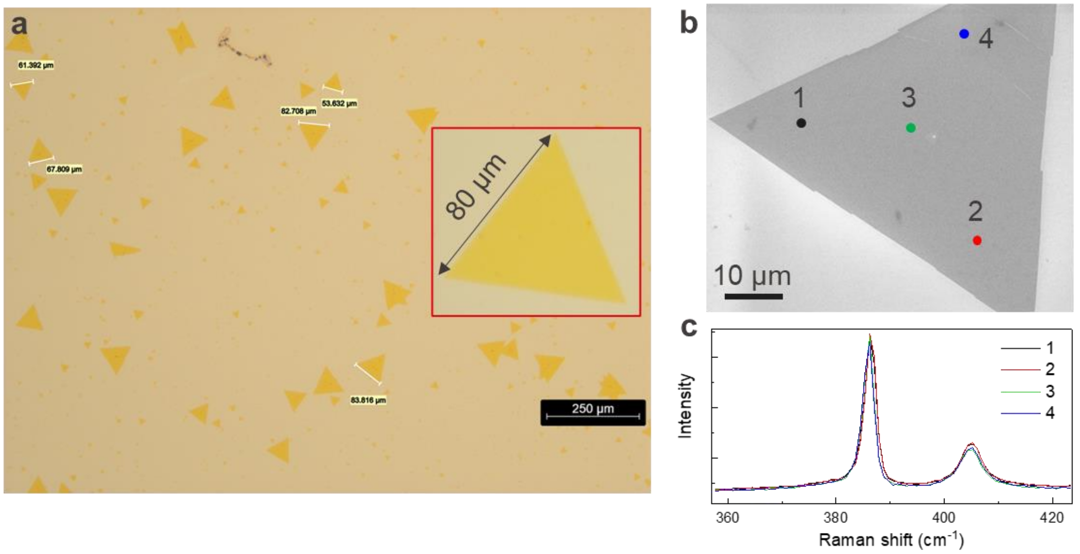

Supplementary Figure 5: Characterization of quality of CVD flakes. (a) Optical image of the triangular monolayer MoS_2 flakes transferred on a SiO_2 substrate and a zoomed-in view of one of the flakes that

appears optically free of micrometer-size cracks. (b) SEM image of the single MoS_2 flake. (c) Raman spectra of the flake.

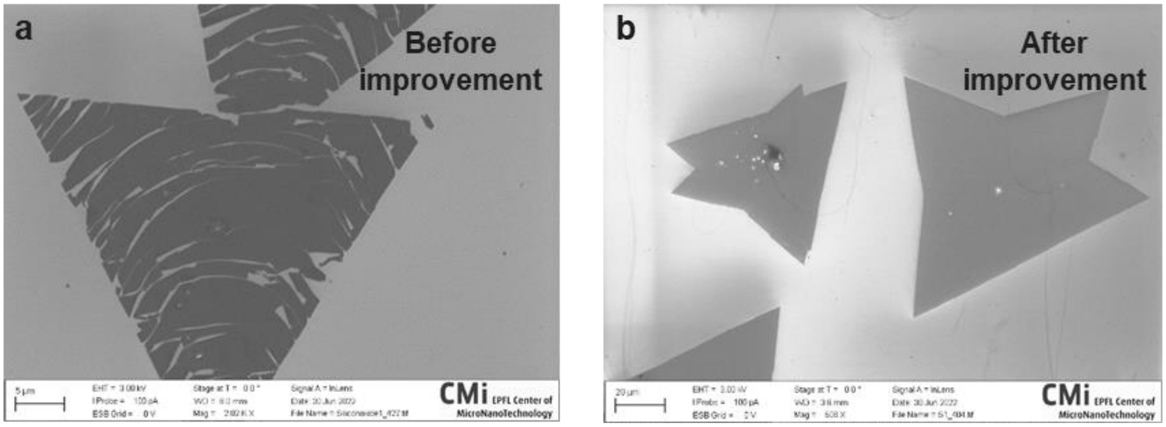

Supplementary Figure 6: The SEM images show (a) a lot of cracks and (b) no cracks before and after improvement in the technique of 2D flake transfer, respectively. The improvement was made in steps d-e in Supplementary Figure 4.

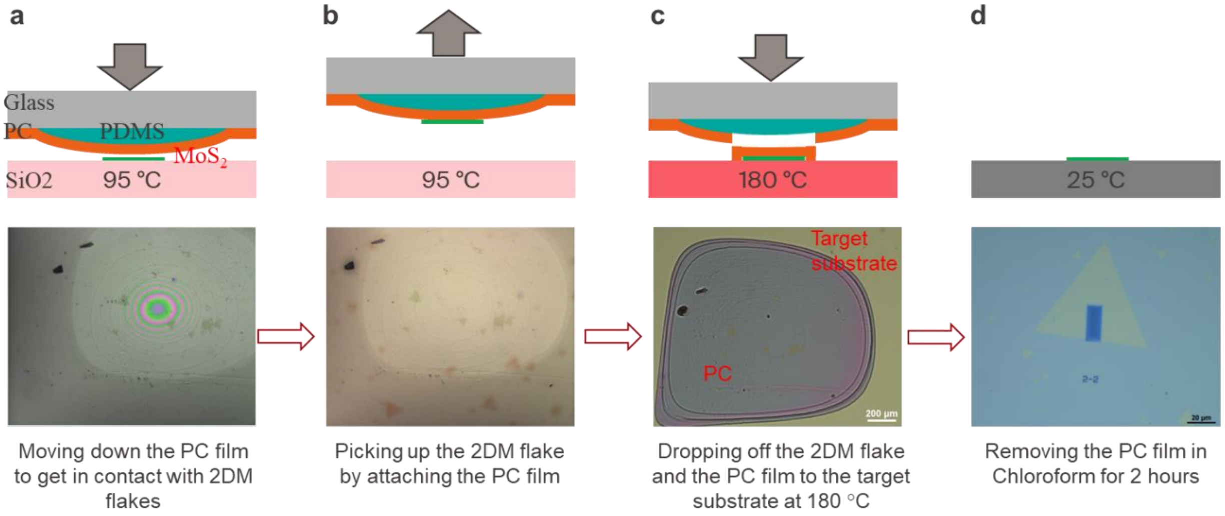

Supplementary Figure 7: Pick-up and transfer of MoS_2 flakes onto a locally patterned substrate. (a) A polycarbonate (PC) film is mounted on top of a PDMS layer with a curved surface. The PC film gets in contact with flakes and the contact area expands with the stage temperature increasing to 95 ºC. (b) The PC film is detached from the substrate and several flakes are peeled off. (c) The PC film with the flakes is transferred onto a target substrate and the PDMS layer is detached at 180 ℃. The PC film in contact with the substrate stays on the substrate together with the flakes. (d) The PC film is dissolved in chloroform for 1 hour. The MoS_2 flake is successfully transferred on the target patterned substrate.

Supplementary Note 4: Transistor fabrication and characterization

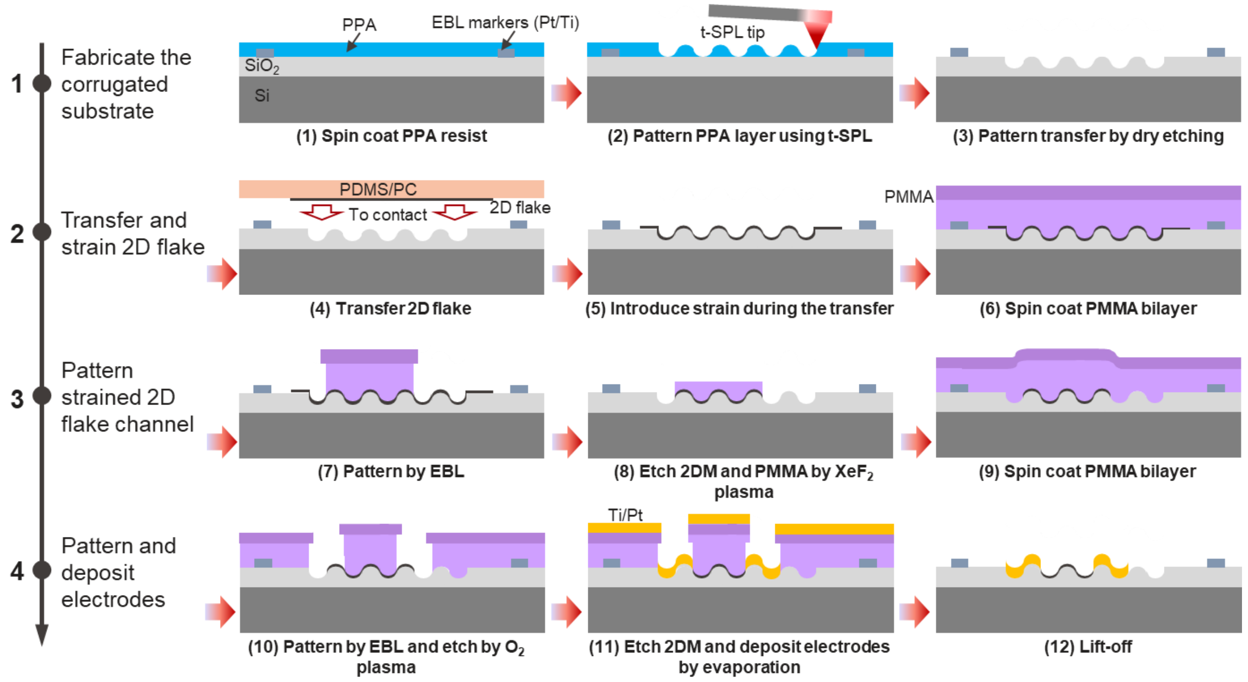

Supplementary Figure 8: Fabrication process of the strained transistors. The process is composed of four steps. The 1st step is to fabricate the biaxial sinusoidal SiO_2 substrate using t-SPL and dry etching. The 2nd step is to transfer the 2D flake on the SiO_2 substrate and to cover the patterned region. Consequently, the strain is introduced into the flake. The 3rd step is to pattern the flake on the nanoengineered substrate. The 4th step is to pattern and deposit electrodes on the strained region and flat region of the same MoS_2 flake using electron beam lithography and PVD, respectively. Note that the strain in the 2D flake is protected under the PMMA resist during the fabrication process.

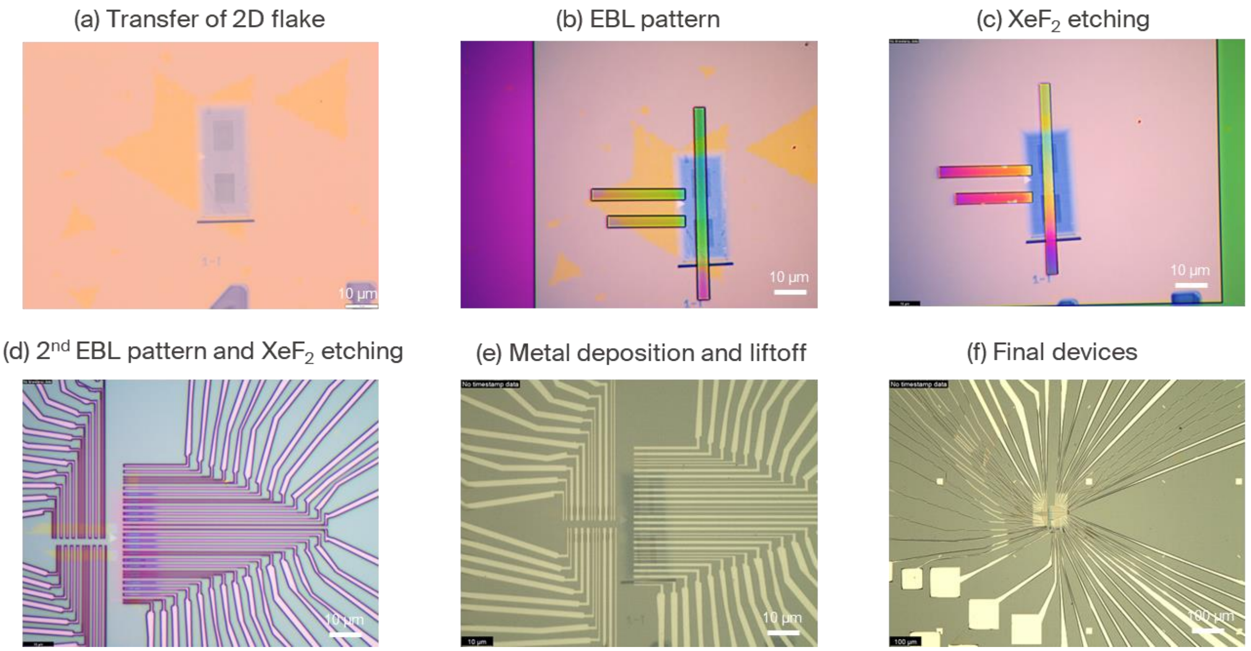

Supplementary Figure 9: Optical images step-by-step along the strained transistor fabrication process using CVD-grown monolayer MoS_2 flakes. (a) Flake transferred onto the nanoengineered substrate. (b) Patterned a PMMA layer on the flake. (c) Patterned the MoS_2 flake protected by the PMMA layer. (d) Pattern of another PMMA layer. (e) Electrode arrays on the strained and flat flakes. (f) Final devices.

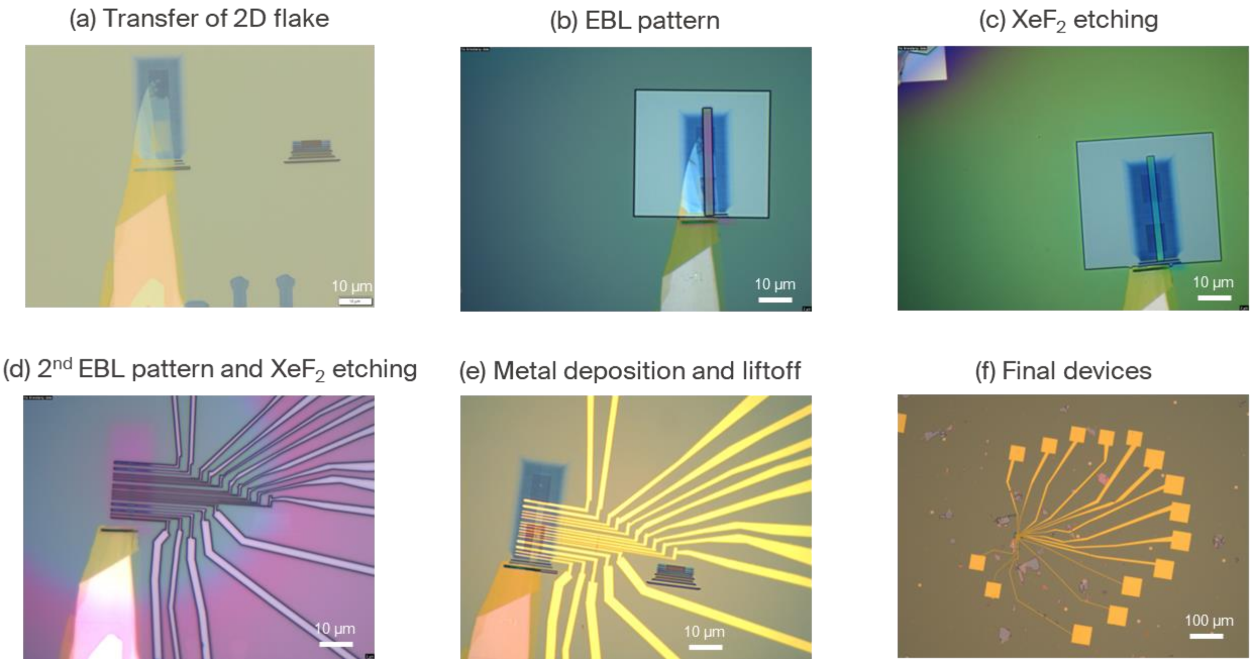

Supplementary Figure 10: Optical images of each step along the fabrication process of the strained transistors made from exfoliated monolayer MoS_2 flakes. (a) 2D material flake transferred onto the pre-

structured substrate. (b) PMMA layer patterning on the flake. (c) The MoS_2 flake etching protected by the PMMA layer. (d) Pattern of another PMMA layer. (e) Electrode arrays fabrication on the strained and flat flakes. (f) Final devices.

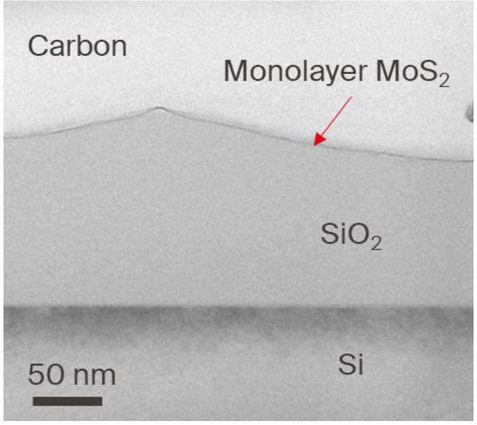

Supplementary Figure 11: Cross-sectional TEM image of the strained transistor's channel showing the monolayer MoS_2 flake follows the wavy SiO_2 substrate with intimate contact.

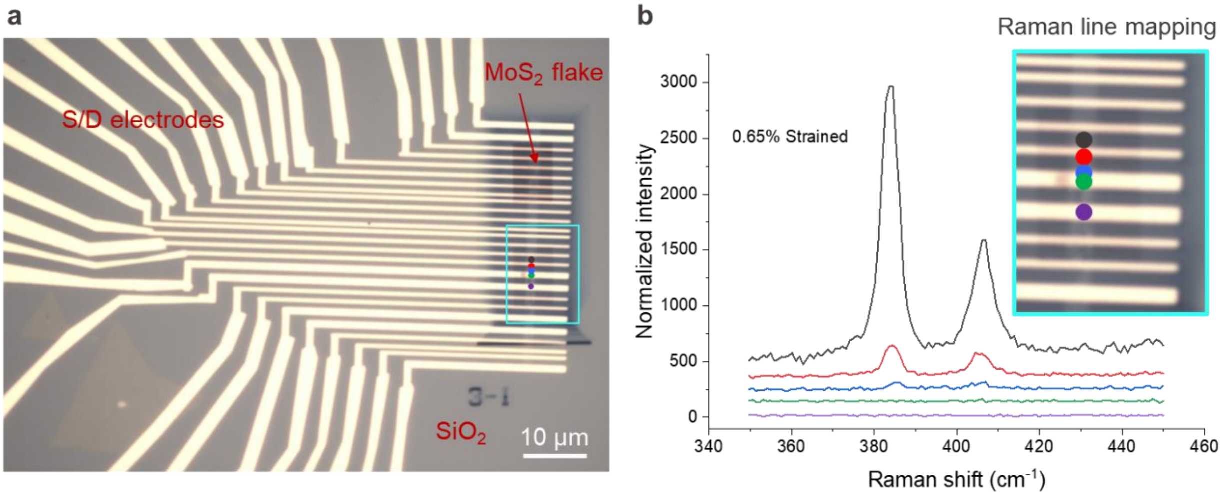

Supplementary Figure 12: (a) Optical image of the strained and the unstrained FETs made from the same monolayer MoS_2 flake. (b) The corresponding Raman line mapping result of the selected area.

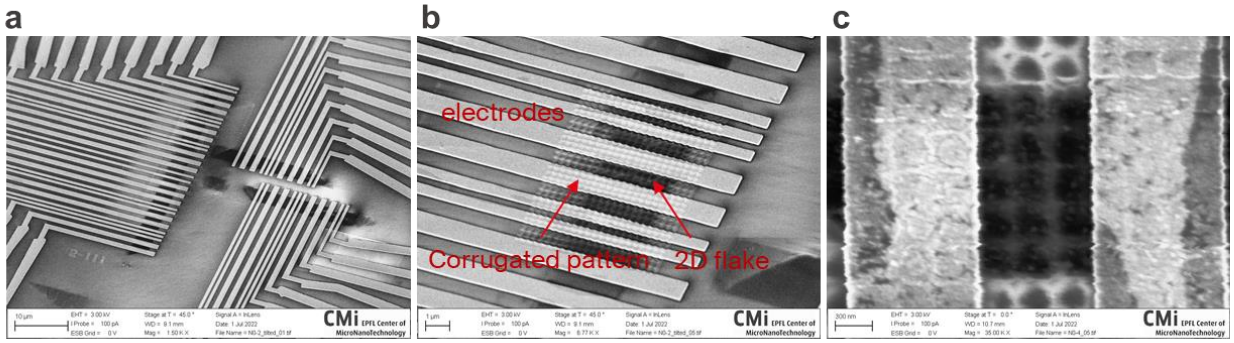

Supplementary Figure 13: SEM images of strained FETs and unstrained FETs made of the same flake with (a) 1.50 kx, (b) 6.77 kx and (c) 35 kx magnifications.

Supplementary Note 5: Strain characterization

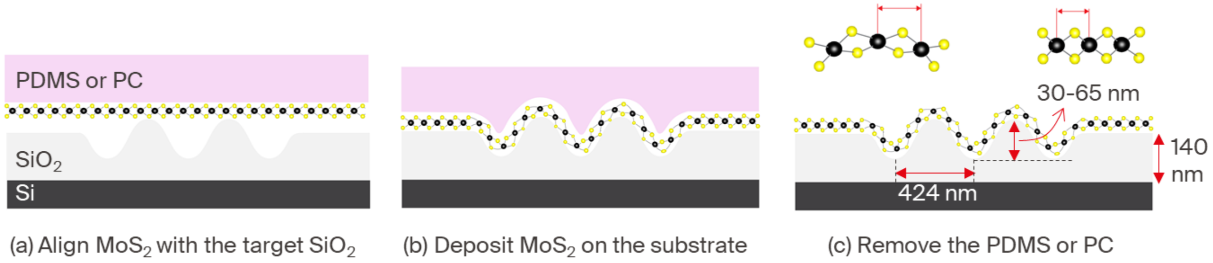

Supplementary Figure 14: The strategy of introducing tensile strain into the 2D flake involves placing the flake on top of a nanoengineered SiO_2 substrate, which causes the flake to elongate along the substrate. (a) The monolayer MoS_2 flake attached on PDMS or PC film is aligned to get in contact with the target substrate. (b) The PDMS or PC film is pressed until it comes into intimate contact with the substrate. (c) The film is removed, leaving the MoS_2 flake adhered to the substrate *via* van der Waals forces.

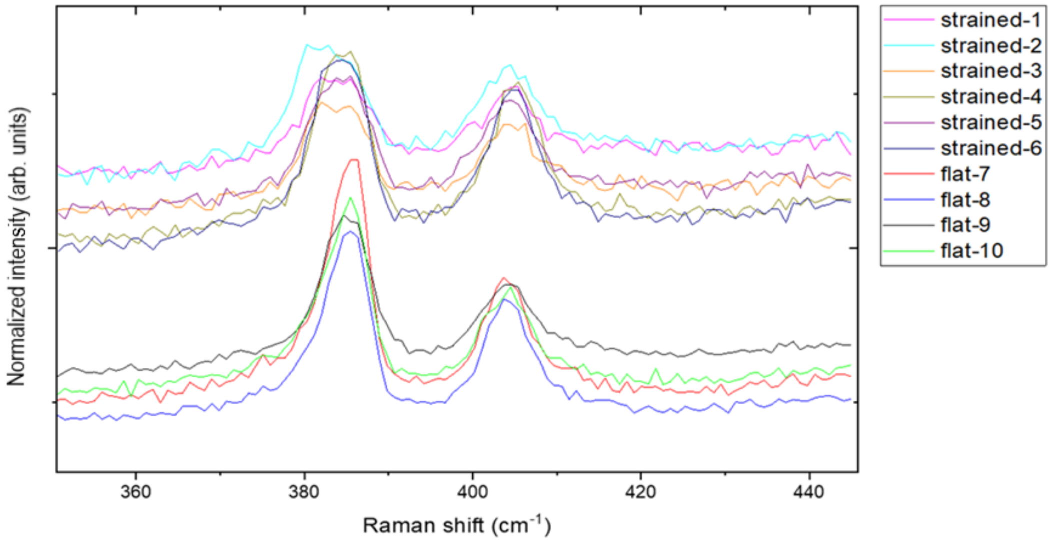

Supplementary Figure 15: Raman spectra of the strained and unstrained FETs made from the same flake corresponding to the data in Fig. 3b.

Supplementary Table 1: Information from Raman spectra of strained MoS_2 and flat MoS_2 FETs

Sample	A_{1g} position, cm^{-1} (micro-Raman)	A_{1g} FWHM, cm^{-1} (micro-Raman)	A_{1g} position, cm^{-1} (TERS)	A_{1g} FWHM, cm^{-1} (TERS)
Strained MoS_2	404.5	9.72	406.7 (top)	7.03 (top)
			406.8 (slope)	7.52 (slope)
			406.6 (bottom)	7.35 (bottom)
Flat MoS_2	404.3	5.81	406.4	6.74

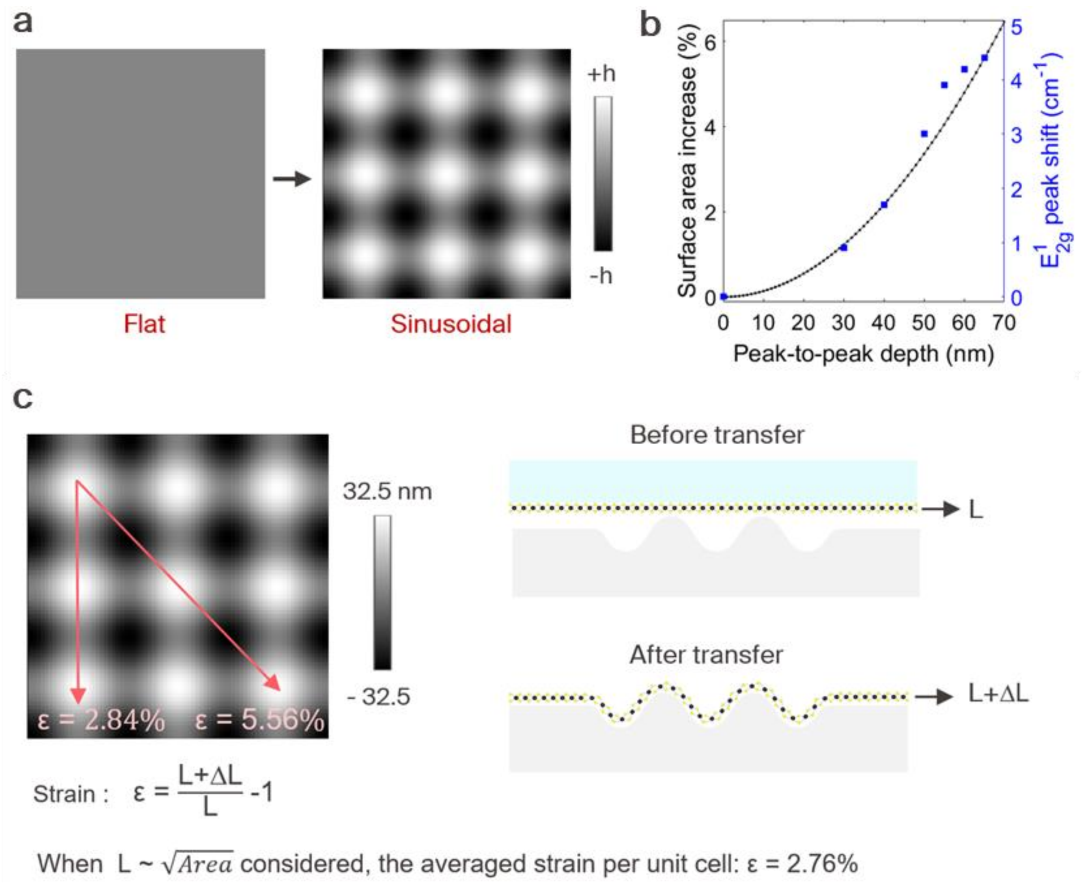

Supplementary Figure 16: (a) Grayscale bitmap illustration of flat and sinusoidal profile, having 2h peak-to-peak depths. (b) Corresponding surface area increase in fixed projection area and E^1_{2g} Raman peak shift comparison. (c) Strain values according to one-directional linear strain formula.

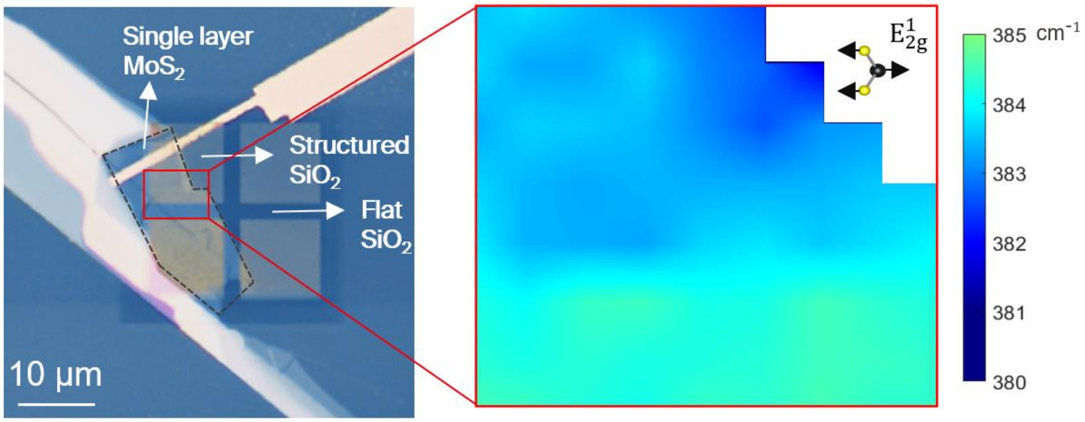

Supplementary Figure 17: Raman mapping of a strained MoS_2 flake. Optical image of the 1L MoS_2 flake transferred onto a nanoengineered substrate (left). Here the parameters of the substrate are the same as those used in the device fabrication except for the area. The flake covers the patterned and flat regions. Raman

mapping of the selected area (right), showing a Raman shift difference of ~5 cm^{-1} in the E^1_{2g} peak position between the strained and the flat areas of the MoS_2. The step size of the Raman map is 1 µm. The data are fitted with a Gaussian function and interpolated in Matlab.

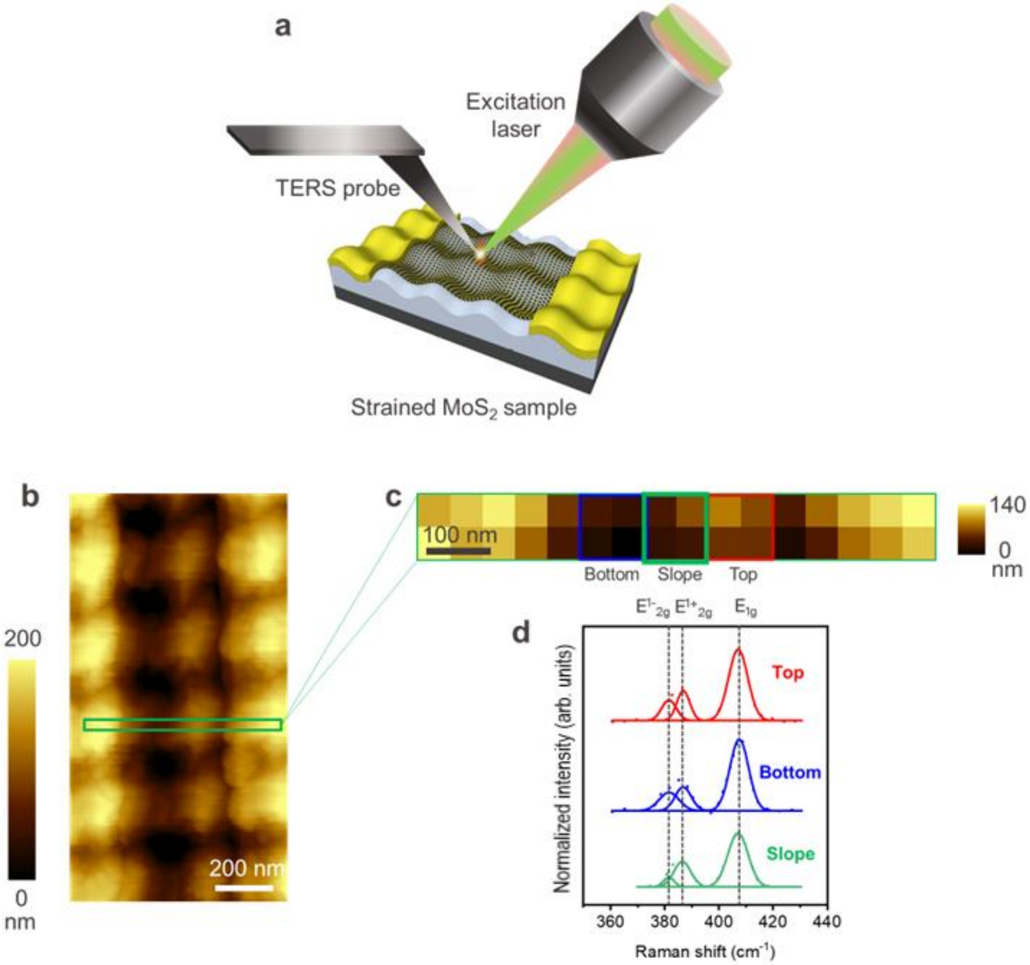

Supplementary Figure 18: Additional data showing the reproducibility of TERS results. (a) Schematic diagram illustrating the experimental setup for TERS measurements of strained MoS_2. (b) AFM topography image showing the nanoengineered structures supporting the monolayer MoS_2 sheet. The region of TERS mapping is highlighted with a green rectangle. (c) AFM topography measured during hyperspectral TERS mapping with a step size of 50 nm. The measured positions are marked with a red square (top region), a green square (slope region) and a blue square (bottom region). (d) Comparison of average TERS spectra measured on the top of the wave (4 pixels, 100 nm × 100 nm), on the bottom and on the slope. Three TERS spectra show nearly identical splitting of the E^1_{2g} peak indicating a similar level of strain in the supported MoS_2 sheet on the top and at the slope of the wave.

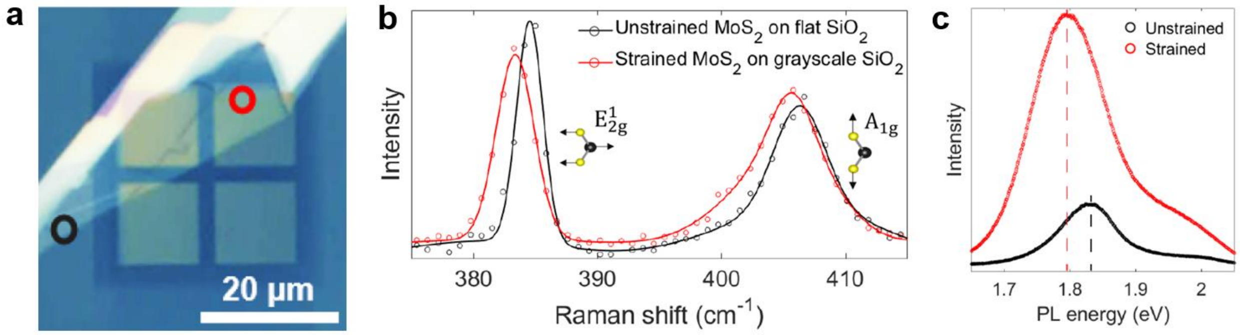

Supplementary Figure 19: (a) Optical image of the exfoliated MoS_2 flake transferred onto a nanoengineered substrate. (b) Micro-Raman spectroscopy characterization of strained 1L MoS_2 on sinusoidal nanopattern modulated in two dimensions and its (c) photoluminescence (PL) characterization to visualize bandgap modulation of MoS_2 monolayer under tensile strain. We observe a ~38 meV shift for a 1.3 cm^{-1} shift in the E^1_{2g} peak position, corresponding to ~132 meV/% strain.

Supplementary Note 6: Electrical performances of strained and unstrained transistors

6.1 Benchmarking Metrics of Strained and Unstrained MoS_2 FETs

The field-effect mobility of the transistor μ_{FE} is calculated using the equation

$$\mu_{FE} = \frac{L_{ch}}{W_{ch}}\frac{1}{C_{ox}}\frac{1}{V_{DS}}\frac{dI_{DS}}{dV_{GS}} = \frac{L_{ch}}{W_{ch}}\frac{1}{\varepsilon\varepsilon_{O}}\frac{1}{V_{DS}}\frac{dI_{DS}}{dE_{GS}} \quad \text{(S1)}$$

where C_{ox} is the capacitance per area, d is the thickness of the dielectric layer, L_{ch} and W_{ch} are the length and width of the transistors, and E_{GS} is the electrical field between the gate and the source.

Conductivity mobility, μ_{con} is also used to estimate the carrier transport property. μ_{con} has the advantage of reflecting the channel material properties and the quality of the channel-dielectric interface.[1] The conductivity mobility, μ_{con} is calculated using the equation

$$\mu_{con} = \frac{1}{qn_sR_{sh}} \tag{S2}$$

$$n_s = \frac{C_{ox}(V_{GS}-V_T)}{q} \tag{S3}$$

where n_s is the carrier density near the source and R_{sh} is extracted from the slope of TLM plots.

Supplementary Table 2: Key parameters of the strained and unstrained FETs

**Strain value**	L_{ch} **[μm]**	I_{on} **[μA/μm]**	V_T **[V]**	R_{sh} **[kΩ/□]**	R_c **(YFM) [kΩμm]**	R_c **(TLM) [kΩμm]**	μ_{FE} **[cm²V⁻¹s⁻¹]**	μ_{con} **[cm²V⁻¹s⁻¹]**
0.90%	1	154	27	7.4	1.2	1.4	**185**	**166**
0.65%	1	116	28	8.9	1.5	1.8	**154**	**146**
0.36%	1	87	30	12.4	2.4	2.1	**101**	**109**
0%	1	32	34	66	3.6	1.2	**26**	**23**

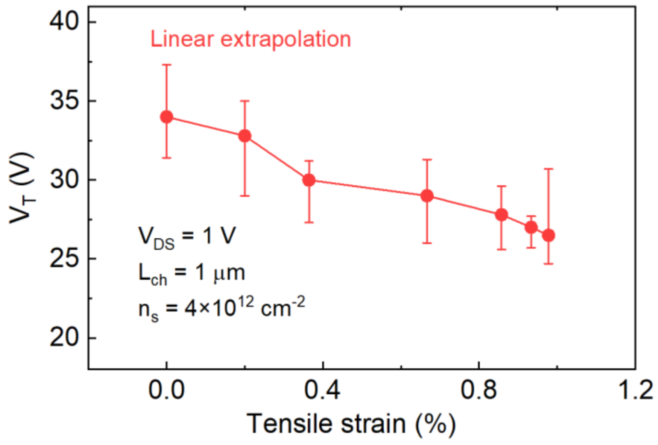

Supplementary Figure 20: Threshold voltage V_T as a function of applied tensile strain from 0% (unstrained) to 1%. The error bars present the maximum and minimum values with the representative points corresponding to the average values.

6.2 Low-Temperature I-V Measurements

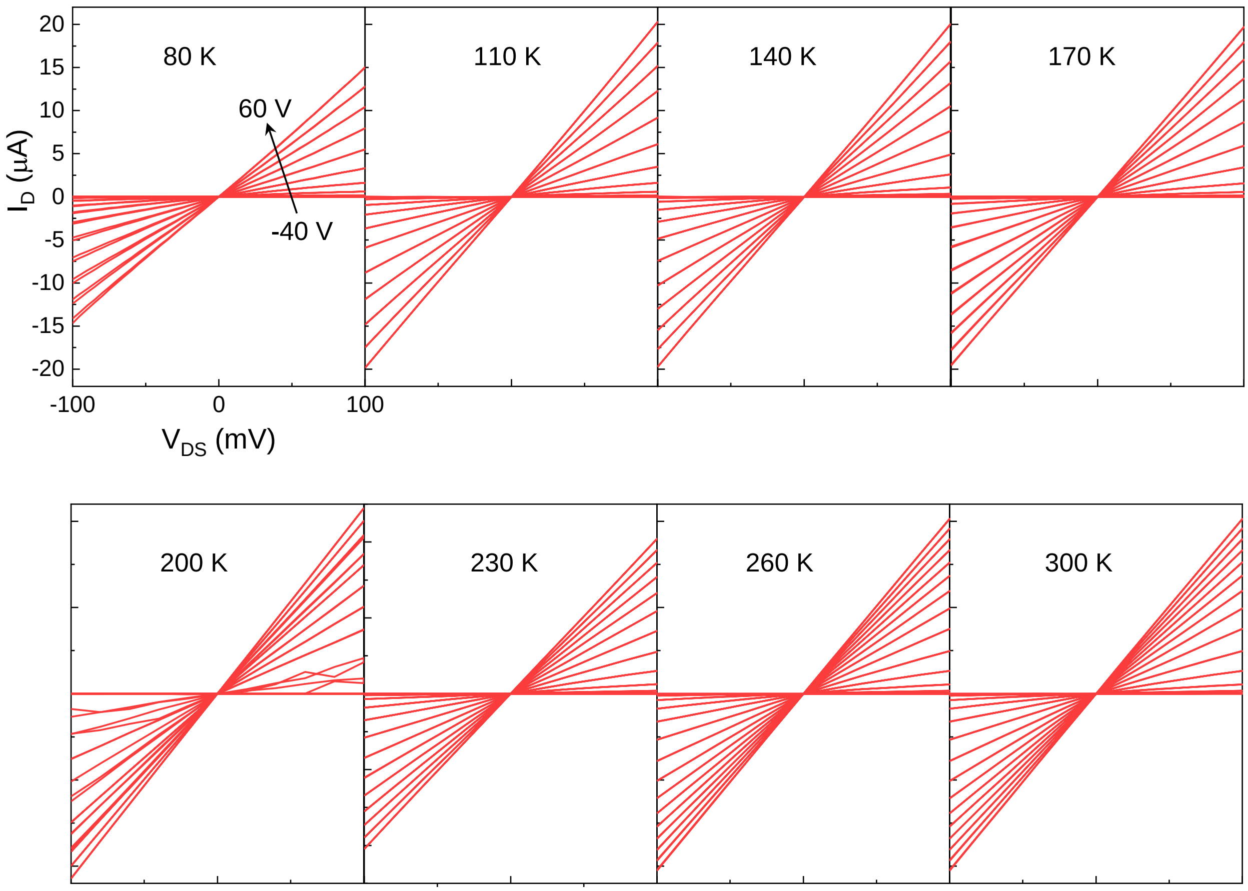

Supplementary Figure 21: Temperature dependence of I_D-V_{DS} curve of the strained MoS_2 FET.

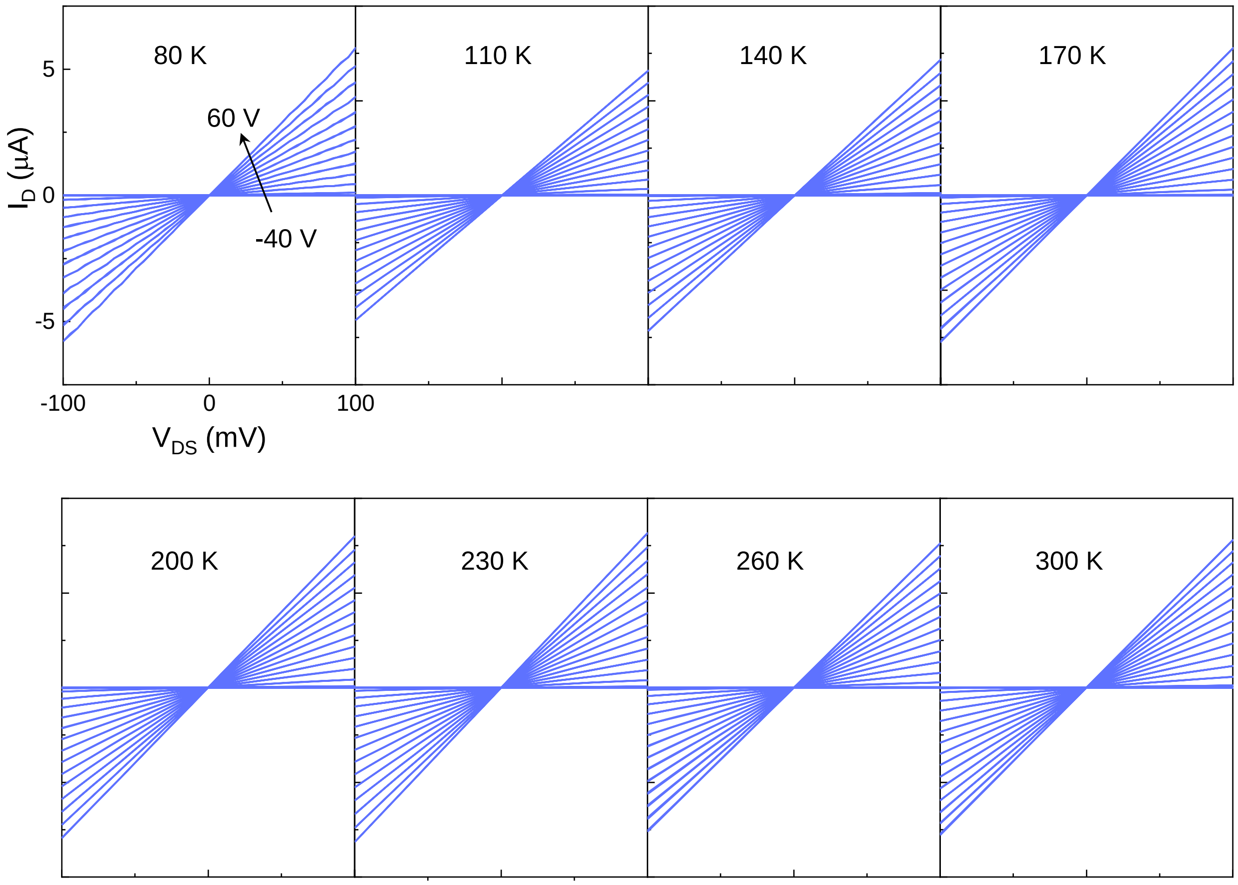

Supplementary Figure 22: Temperature dependence of I_D-V_{DS} curve of the unstrained (flat) MoS_2 FET.

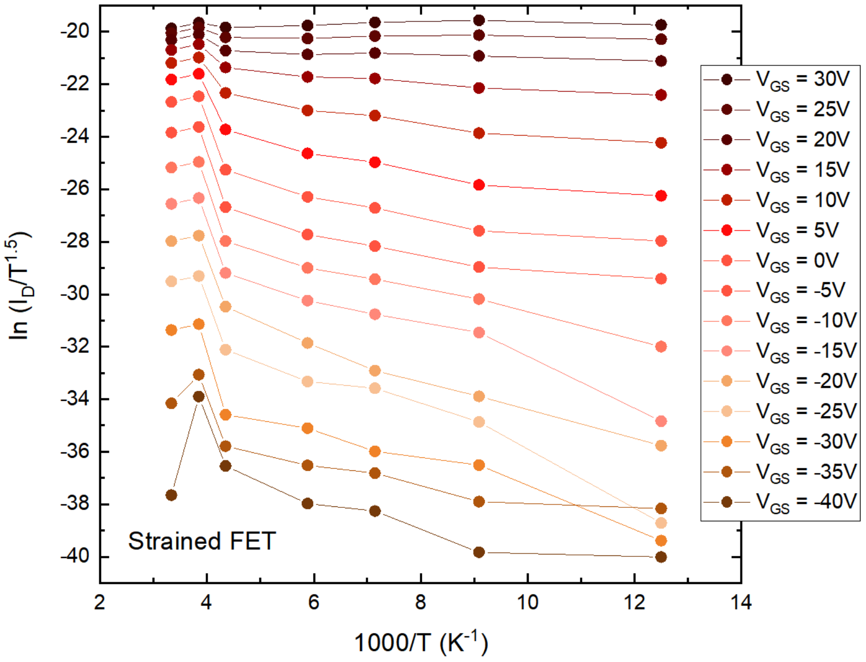

Supplementary Figure 23: Arrhenius plots at different values of V_{GS} in the strained FET with a channel length of 1 μm.

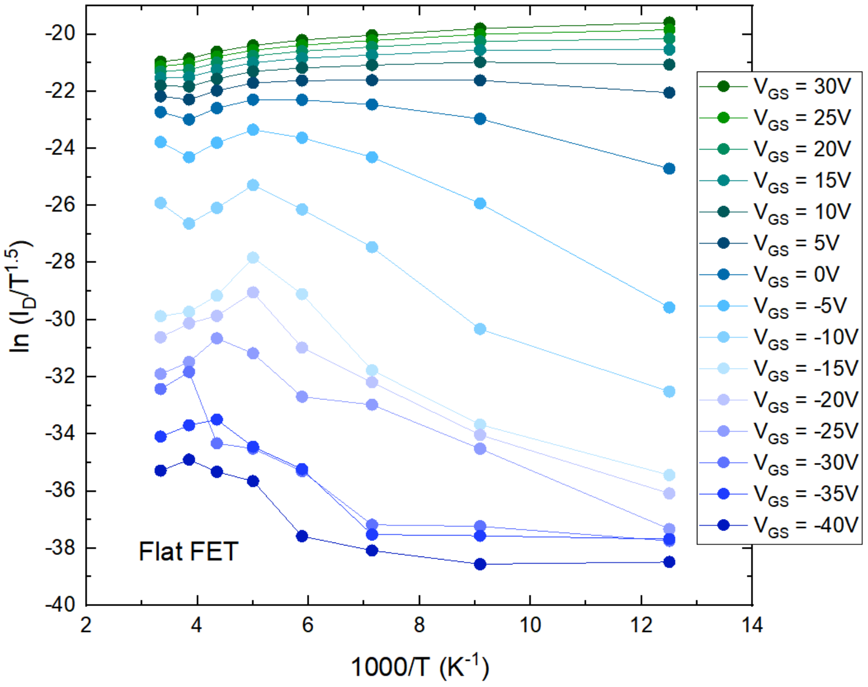

Supplementary Figure 24: Arrhenius plots at different values of V_{GS} in the flat FET with a channel length of 1 μm.

6.3 Extraction of Schottky Barrier Height

Using the equation $I_D = AT^{1.5} exp\left({}^{q\emptyset_{SB}}/_{k_BT}\right)\left[1 - exp\left({}^{qV_{DS}}/_{k_BT}\right)\right]$, the effective Schottky barrier height $\emptyset_{SB}$ is extracted. In the equation, A is Richardson's constant, k_B is Boltzmann's constant, q is the electronic charge, T is the temperature. Following the conventional thermionic emission theory, the Schottky barrier height $\emptyset_{SB}$ is determined by the linear fitting of Arrhenius plots at various back gate voltages.[2–4]

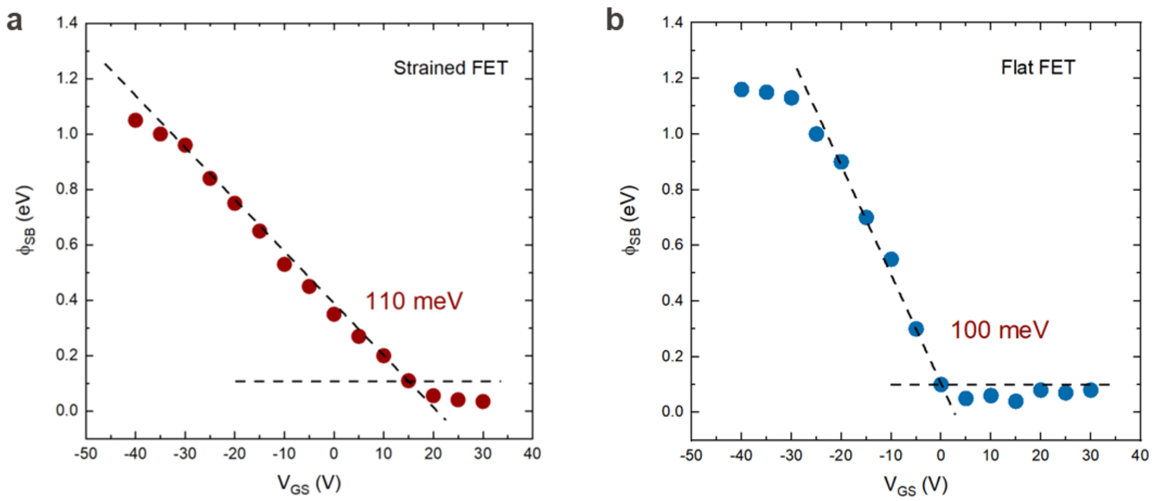

Supplementary Figure 25: Schottky barrier height (SBH) for (a) a strained transistor and (b) a flat transistor on the same flake. The extracted value of the SBH can change by about ±15% for a reasonable variation of the extraction region. Strained and flat FETs both have channel lengths of 1 μm.

6.4 Analysis of Sheet Resistance and Contact Resistance

6.4.1 Transfer length method (TLM)

The total resistance per unit of channel width R_{tot} (in Ω·m) of the transistors in the TLM structure consists of the channel resistance per unit of channel width R_{ch} (in Ω·m) and the contact resistance per unit of channel width R_c (in Ω·m), is expressed as:

$$R_{tot} = R_{ch} + 2R_c = R_{sh} \cdot L_{ch} + 2R_c \quad \text{(S4)}$$

where R_{sh} (in Ω/□) is the sheet resistance, L_{ch} is the channel length. The total resistance, R_{tot} can be plotted as a function of the channel length L_{ch}. Therefore, by linearly fitting the curve, the sheet

resistance and the contact resistance can be readily obtained from the slope of the fitting line and from the intercept with the vertical axis when $L_{ch} = 0$, respectively. Supplementary Figure 26 shows the data.

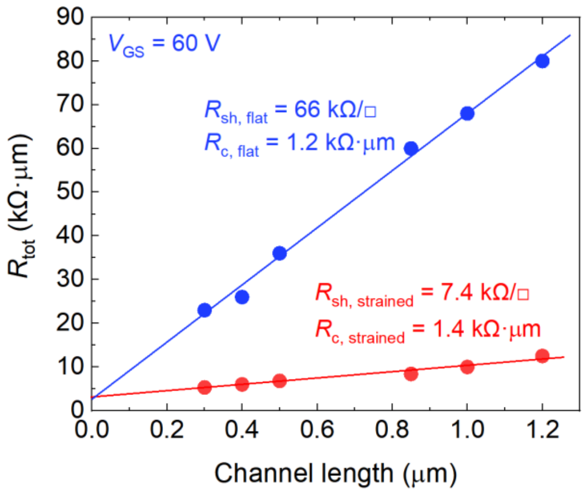

Supplementary Figure 26: Sheet resistance (R_{sh}) and contact resistance (R_c) extractions using the transfer-length method (TLM) when V_{GS} is 60 V.

6.4.2 Y-function method (YFM)

To estimate the contact resistance of the flat and strained MoS_2 FETs, we employ the Y-function method (YFM).[5] According to YFM, the Y-function can be expressed as

$$Y = \frac{I_D}{\sqrt{g_m}} = \sqrt{\mu_0 C_{ox} V_{DS} \frac{W_{ch}}{L_{ch}}}(V_{GS} - V_T) \quad \text{(S5)}$$

where g_m is the transconductance, μ_0 is the low field mobility, C_{ox} is the gate dielectric capacitance, V_T is the threshold voltage, W_{ch} is the channel width, and L_{ch} is the channel length. In strong inversion, Y should be linear in gate voltage with intercept and slope giving the threshold voltage V_T, and the low field mobility parameter μ_0, respectively. Here, V_T is extracted from the $I_D/g_m^{0.5}$ curve. Using linear fitting of the plot of Y-function with respect to V_{GS}, μ_0 can be extracted. The expression of mobility attenuation factor θ due to contact resistance, surface roughness, and phonon scattering as a function of V_{GS} can be written as

$$\theta = \left[{I_D}\Big/{\left(g_m(V_{GS} - V_T)\right)} - 1 \right] \Big/ (V_{GS} - V_T) \quad \text{(S6)}$$

The mobility attenuation factor includes the effects of the S/D series resistance and can be expressed as

$$\theta = \theta_0 + 2R_c\mu_0 C_{ox} {}^{W_{ch}}/_{L_{ch}}, \tag{S7}$$

where θ_0 is the intrinsic mobility attenuation factor.[6] For 2D transistors, θ_0 is considered negligible. As a result, the contact resistance R_c can be calculated from θ,

$$2R_c = \theta/(\mu_0 C_{ox}\frac{W_{ch}}{L_{ch}}). \tag{S8}$$

In strong inversion, this function (θ versus V_{GS}) is expected to be a constant equal to the value of the mobility reduction coefficient. Using Eqs. (S5)-(S8), we estimate R_c of the strained and flat FETs.

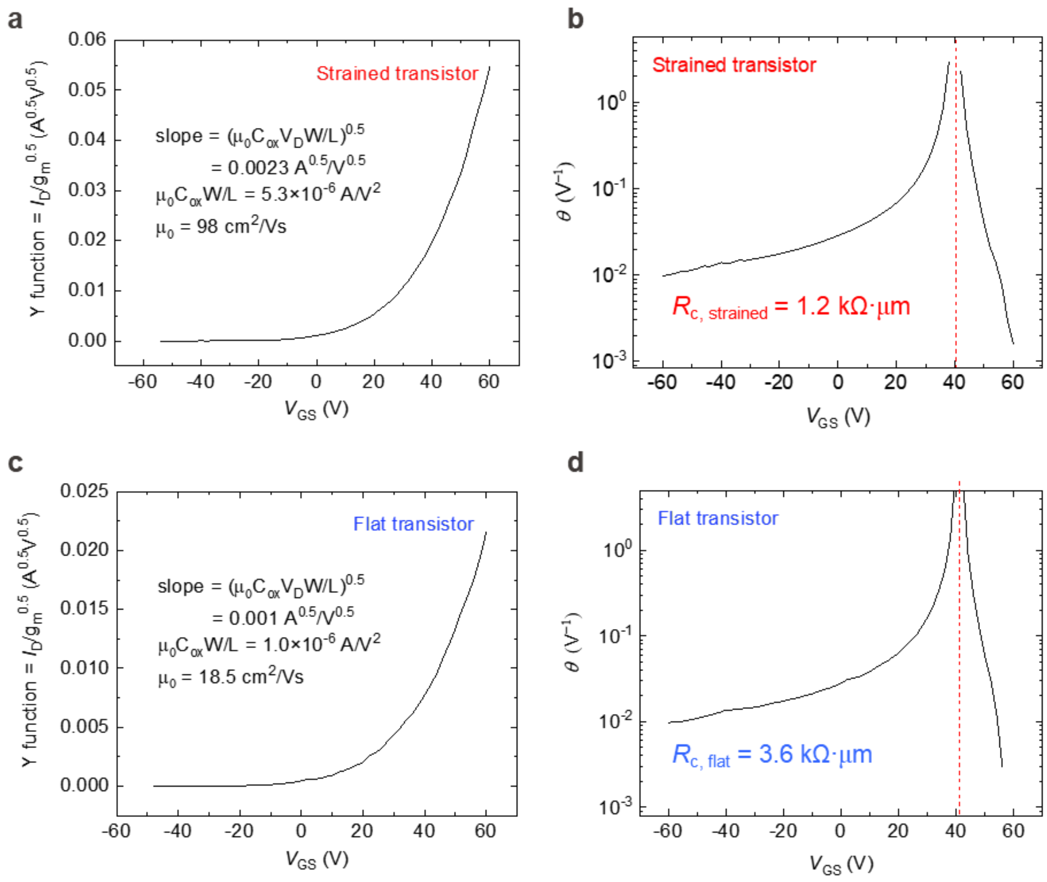

Supplementary Figure 27: Calculation of contact resistance using the Y-function method (YFM). (a, c) Y function as a function of V_{GS} for the strained and flat FETs, respectively. (b, d) Mobility attenuation factor θ as a function of V_{GS} for the strained and flat FETs, respectively.

6.5 Reproducibility of the measurements

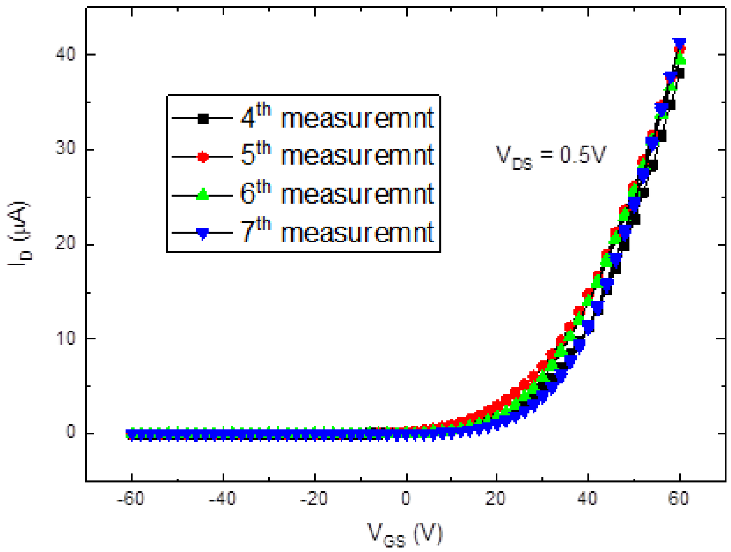

Supplementary Figure 28: I_D-V_{GS} transfer characteristics of the strained FET measured in air repeatedly within a few minutes to show the stability of the measurements. The first three measurements are not considered since they act as a thermal annealing treatment. Once the water molecules/air on the surface of the device are removed, the measurement gets stable.

6.6 Stability of the devices over time

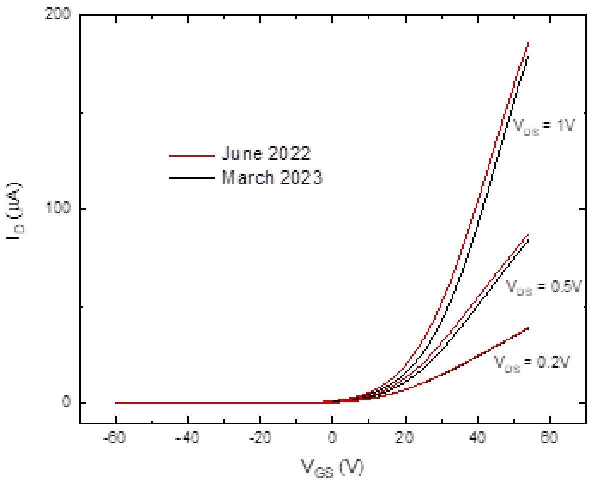

Supplementary Figure 29: The I_D-V_{GS} transfer characteristics of the strained FET measured in air at different time. The sample was stored in vacuum.

Supplementary Table 3: Comparison of performance of strained MoS_2 FETs

Refs	Material<br>Thickness<br>Method	Strain type	Strain value [%]	Raman shift [cm^{-1}]	Carrier density [cm^{-2}]	Strained FET		Unstrained FET [$cm^2V^{-1}s^{-1}$]
						Mobility [$cm^2V^{-1}s^{-1}$]	Enhance ment	
**Experimental results**								
**[7]**	MoS_2<br>1L<br>CVD grown	Uniaxial tensile strain	0 ~ 0.7	E^1_{2g}: 1.61	1.1×10^{13}	14<br>for 1L MoS_2 at 0.7% strain	**2x**	7
**[8]**	MoS_2<br>1L<br>CVD grown	Uniform biaxial tensile strain	0.7	E^1_{2g}: 3.3	NA	15.94	**1.8x**	8.71
**[9]**	MoS_2<br>1L<br>CVD grown	Biaxial tensile strain	0.23	NA	NA	9.1<br>for 1L MoS_2 at 0.23% strain	**1.7x**	5.4
**[10]**	MoS_2<br>1L and 2L<br>Exfoliated	Uniaxial tensile strain	0.73-1.7	E^1_{2g}: 3.36-7.82	2×10^{12}	127<br>for 1L MoS_2 at 0.87% strain<br>82<br>for 2L MoS_2 at 1.36% strain	**2.52x (1L)**<br>**1.64x (2L)**	50.5
**[11]**	MoS_2<br>1L and 3L<br>Exfoliated	Biaxial tensile strain	NA	0	9×10^{12}	448<br>for 1L MoS_2<br>900<br>for 3L MoS_2	**20x (1L)**<br>**100x (3L)**	20<br>(1L)
**[12]**	MoS_2<br>1L<br>CVD grown	Local biaxial tensile strain	0.2−1.3	E^1_{2g}: ~6.5<br>A_{1g}: ~3.5	(0.8−1.8) $\times10^{12}$	32	**60x**	0.5
**[13]**	MoS_2<br>1L<br>Exfoliated	Tensile strain	~0.7	NA	7.8×10^{12}	~1150<br>for 1L MoS_2 at 0.7% strain	**100x**	12.5
**Our work**	MoS_2<br>1L<br>Exfoliated and CVD grown	Multiaxial tensile strain	0 ~ 1	E^1_{2g}: 4.4	3.7×10^{12}	185<br>for 1L MoS_2 at 0.9% strain	**8x**	23
**Theoretical calculation results**								
**[14]**	MoS_2<br>1L	Biaxial tensile strain	5	NA	1×10^{12}	45	**1.53x**	30
**[15]**	MoS_2<br>1L	Biaxial tensile strain	0.4	NA	1×10^{12}	NA	**1.05x**	NA
**[16]**	MoS_2<br>1L	Uniaxial strain	2	NA	NA	0.9	**6x**	0.15
**Our work**	MoS_2<br>1L	Uniform tensile strain	0 ~ 1	NA	5×10^{12}	485<br>for 1L MoS_2 at 1% strain	**8.46x**	57.3

Supplementary Note 7: Electrical field distribution in strained FETs

Due to the variation of the thickness of the patterned SiO_2, the distribution of the electrical field on the strained MoS_2 channel is not homogenous as shown in Supplementary Figure 30. The analytical calculation of the capacitance at specific dielectric heights of the strained transistor is calculated using the equation:

$$C_{ox} = \frac{1}{{V_{GS}}^2}\, \varepsilon_0\, \varepsilon \sum (E(y))^2\ \Delta y \quad \text{(S9)}$$

where V_{GS} is the voltage of back gate, ε is the dielectric constant, E(y) is the electrical field at the position y. Along the longitudinal direction, extending from the bottom to the dielectric surface, and in real-life, from the gate to the FET channel, we initially simulated the electric field distribution using COMSOL Multiphysics (version 6.0) with electrostatics physics modeling. Subsequently, we extracted the electric field norm distribution at specific positions of sinusoidal design modulated in two dimensions *f(x,z)*, such as dielectrics with heights of 110 nm, 140 nm, and 170 nm, and performed capacitance calculations in MATLAB (version R2020b) utilizing the aforementioned formula. Furthermore, we compared our capacitance calculations obtained through combined simulation and analytical approaches with parallel plate capacitance assumptions at heights of 110 nm, 140 nm, and 170 nm, and observed deviations in results of less than 3%. Local capacitances in the sinusoidal design exhibited a proportional relationship to the parallel plate approach, with the mean values of calculated gate capacitance across different heights equating to the capacitance at 140 nm.

Finally, when the Maxwell capacitance of our grayscale design in COMSOL Multiphysics simulation was computed, it was only 3.5% higher than that of the flat design, as shown in Figure S31.

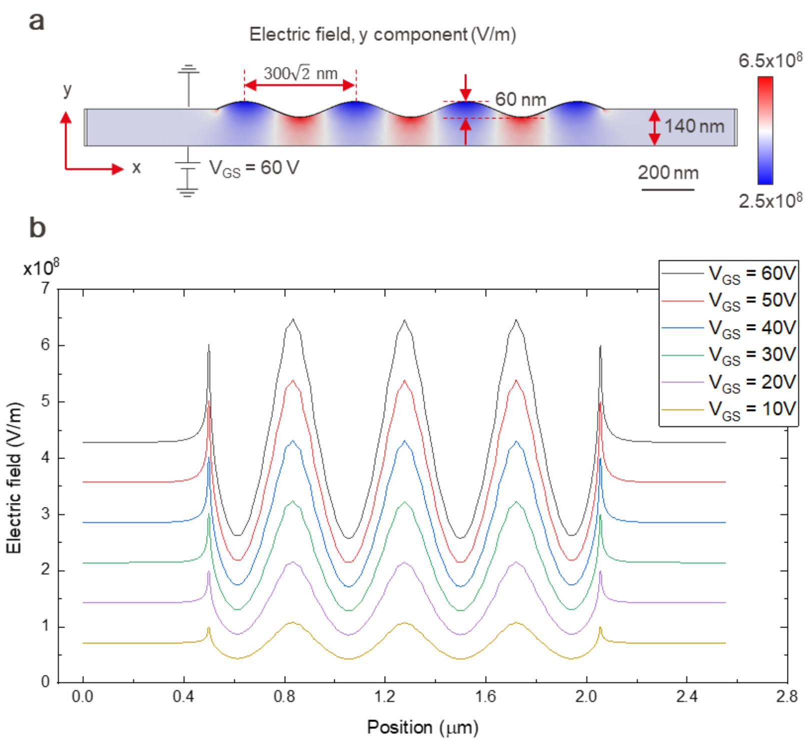

Supplementary Figure 30: (a) Electrical field distribution along the apex position in wavy surface capacitor. (b) Gate voltage dependent electrical field distribution along the transistor channel.

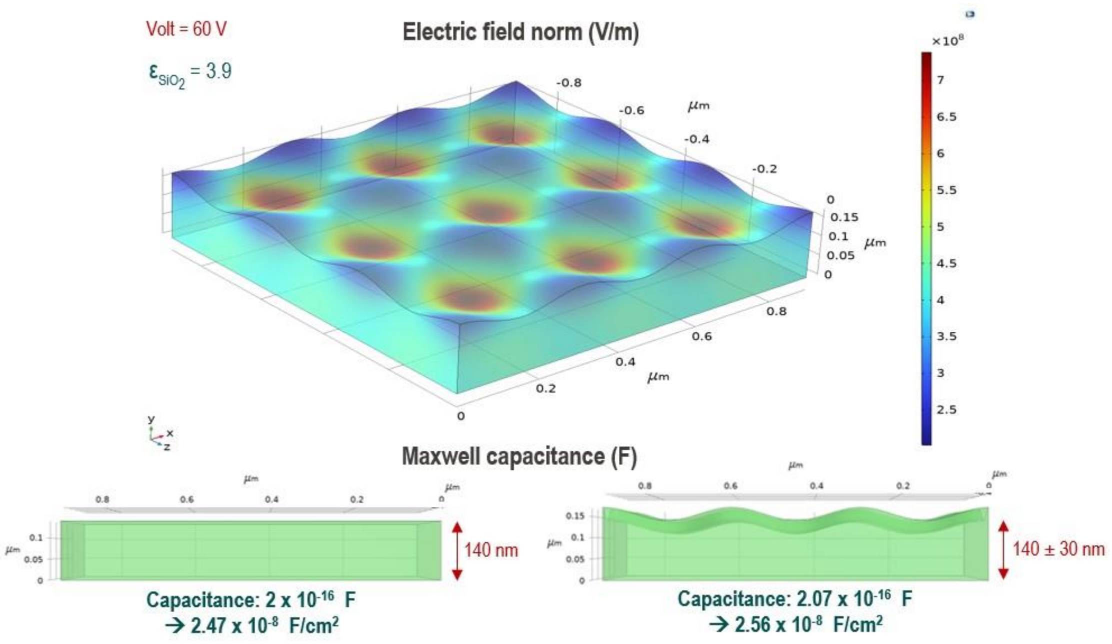

Supplementary Figure 31: Electrical field distribution (top) in structured SiO_2 with sine waves having 300 nm pitch and 60 nm peak-to-peak amplitude and capacitance values (bottom). These simulations represent our flat and wavy designs used for unstrained and strained 2D FETs. The Maxwell capacitance of the wavy design is only 3.5% higher than that of the flat one.

Supplementary Note 8: First-principles calculation on tensile strain enhanced carrier mobility

In the main text, we have provided an overview of the computation of mobilities, emphasizing the reliance on density-functional perturbation theory (DFPT) and the first-principles parametrized model as detailed in reference.[17] Here, we explain the procedure in more details and we further justify the use of this model.

First-principles model for phonon-limited mobility vs strain

Under the assumption that the change in mobility under stain in mainly ruled by the change in the valley energy separation, ΔE_{KQ}, the main goal becomes to relate tensile strain, ΔE_{KQ} and mobility enhancement. Using the experimental structure[18] without relaxing the atomic positions, we compute a reference value for the valley energy separation in the unstrained material: ΔE_{KQ} = 34 meV. Our objective is then to study how ΔE_{KQ} changes under the influence of strain. However, when performing strain calculations in density-functional theory (DFT), it is essential to relax the out-of-plane atomic positions. These positions are directly linked to the energy difference between the K and Q valleys, a factor we have established as crucial for mobility. Importantly, even before any strain is applied (i.e., in the flat system), this relaxation process leads to an overestimation of $\Delta E_{KQ.}$ Acknowledging Kohn-Sham DFT's limitations in predicting band structures,[19] we address this overestimation by comparing ΔE_{KQ} values between the unrelaxed experimental structure and the relaxed one. We assume that the calculations (after relaxation) consistently overestimate ΔE_{KQ} by the same amount for each strain value. Consequently, we proceed to relax the structure at different strain values, ranging from 0 to 2%. This allows us to track how ΔE_{KQ} evolves under strain (see Supplementary Figure 32). In the end, to ensure that our results align with the experimental structure (with an offset of 34 meV), we apply a consistent shift to the ΔE_{KQ} for each

strain. This is how we obtain the data Fig. 5a in the main text. Finally, correcting electron-phonon interactions with the model,[17] we solve the Boltzmann transport equation (BTE) at each shift to obtain mobility as a function of valley positions, following the approach in reference.[17] In principle, one could compute the mobility for each strain fully from first-principles (i.e., without the model). However, this approach is expensive. More importantly, the mobility results would be inaccurate due to the necessity of relaxation of atomic positions. As explained, this relaxation is crucial for obtaining physical phonons and electron-phonon interactions but brings an incorrect valley-energy separation. This leads to an overestimation of the mobility, as the Q valleys become unreachable for electrons even in the absence of strain, resulting in the complete suppression of intervalley couplings.

Discussion on fully first-principles phonon-limited mobility calculations

To further investigate the strain effect in terms of transport, we focused on a fully first-principles and systematic comparison of the relevant properties for the strained system at 2% tensile strain with respect to the unstrained (flat) material. Supplementary Figure 33 illustrates the valleys shift mechanism induced by the strain values investigated in terms of band structure and density of states. Here the crystal structure of reference is the equilibrium one obtained from DFT, i.e., starting from the experimental one and consistently relaxing (both lattice parameters and atomic positions) for the flat and the strained cases. We set a target electrostatic n-doping of $N_d = 5\times10^{12}$ cm^{-2} and room temperature conditions (i.e., T=300 K). For the electronic properties, the same considerations expressed in the main text (using the experimental crystal structure, unrelaxed) are valid, with the key difference being a much larger energy separation between the K and Q valley even in the flat system. However, straining a generic material is expected to affect not only its electronic band structure but also phonons and electron-phonon interactions. Similar to the

electronic band structure, phonons are also considerably affected by the applied strain, as shown in Supplementary Figure 34. The major modifications concern the out-of-plane acoustic modes (ZA) and the optical homopolar mode. In terms of electron-phonon coupling (EPC), the analysis becomes more complex (see Supplementary Figure 35). Acoustic phonons are known to be the main contributors to transport: not only their coupling with electrons is the most relevant, but also, at room temperature, these modes are the only ones that significantly populated. To confirm this, we solve the Boltzmann transport equation (BTE) by including only the three low-energy modes corresponding to the acoustic displacements, and we find that these modes contribute almost 80% of the total value of the mobility. Note that this is just a qualitative check: the BTE solution cannot be trivially decoupled as a sum of contributions from each mode. Thus, we focus on the EPC plots only for the three acoustic modes. The main variation concerns the third phonon mode (energetically ordered, and corresponding to the ZA) which, after strain, couples to electrons up to one order less strongly (depending if one considers the initial electronic state to be in the K or K' valley). The other variations are subtle and non-intuitive due to the crossing of low-energy phonon modes. In general, one can observe that intervalley scattering is suppressed in the strained system, as expected, where mainly long-wavelength and zone edge phonons still couple with electrons, thus potentially hindering transport. In conclusion, the reduction in coupling with the ZA phonons results in an overall mobility enhancement under strain of about 15%, i.e., from 450 cm^2/Vs to 520 cm^2/Vs. In other words, these modifications lead to a mobility increase of only 15% for 2% strain, far from the enhancement factor found in experiments. The reason is that, as previously highlighted, the equilibrium structure corresponds to a large energy separation between the K and Q valleys of 220 meV already in the unstrained case. As a consequence, even if the Q valleys fall in the thermal layer, they are not expected to be significantly populated, and thus they will contribute scarcely to transport in either case, strained or unstrained.

These results underpin that the valley-shift as the primary mechanism to explain the experimental results and support the use of the first-principles model, as explained in the main text, with the recommendation that is essential to use as a starting point the experimental structure with a separation between the K and Q valleys as close as possible to the real one.

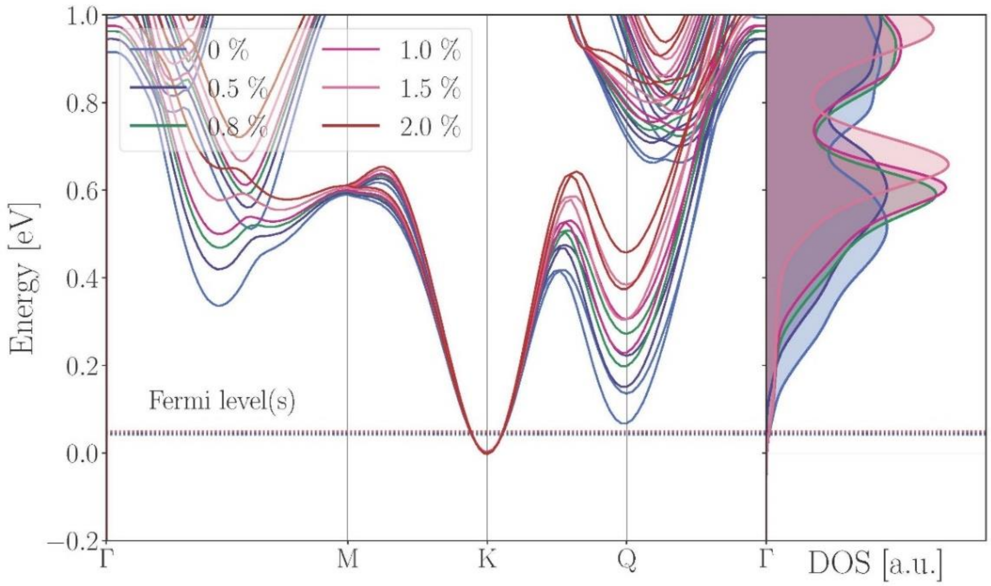

Supplementary Figure 32: Electronic band structure and density of states for the different strains considered in this work. The offset is chosen to be the bottom of the conduction band for each case, and the Fermi level accounting for doping is shown.

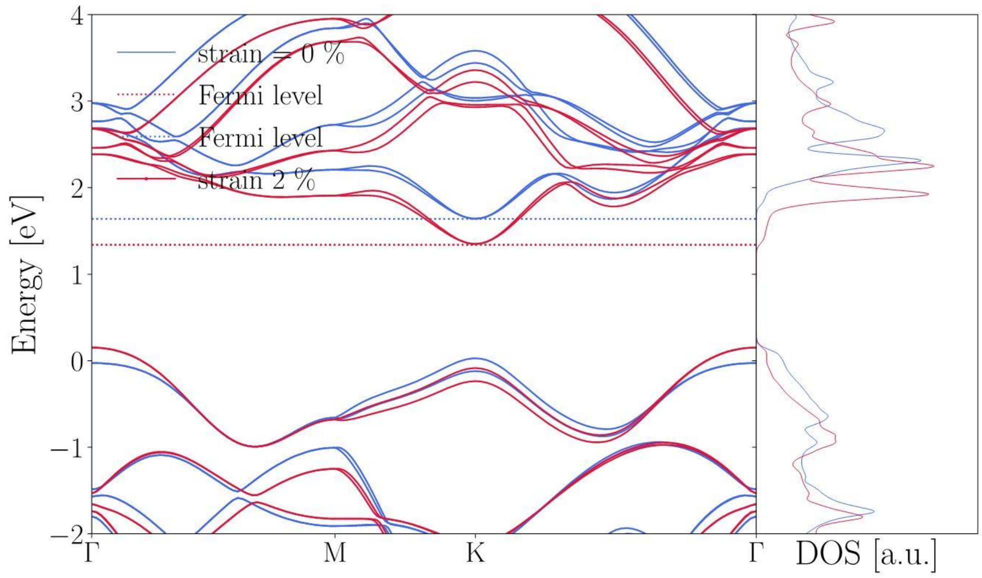

Supplementary Figure 33: Electronic band structure and density of states for the unstrained system (blue) and the 2% tensile strained one (red). The zero of energy is set at the top of the valence band for each case, the Fermi level, accounting for doping and electronic temperature, is also shown.

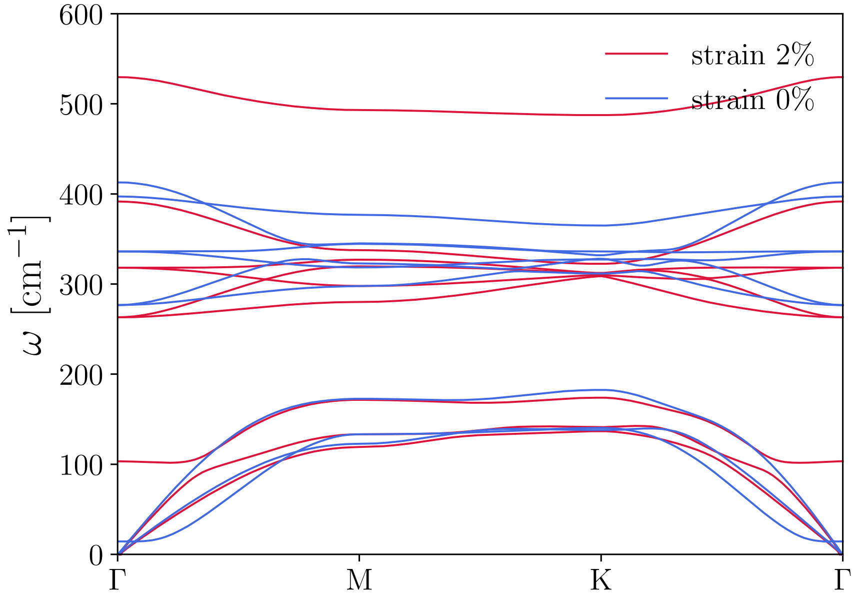

Supplementary Figure 34: Phonon dispersion curves for unstrained MoS_2 (blue) in comparison with the strained case (2% tensile uniform strain) (red).

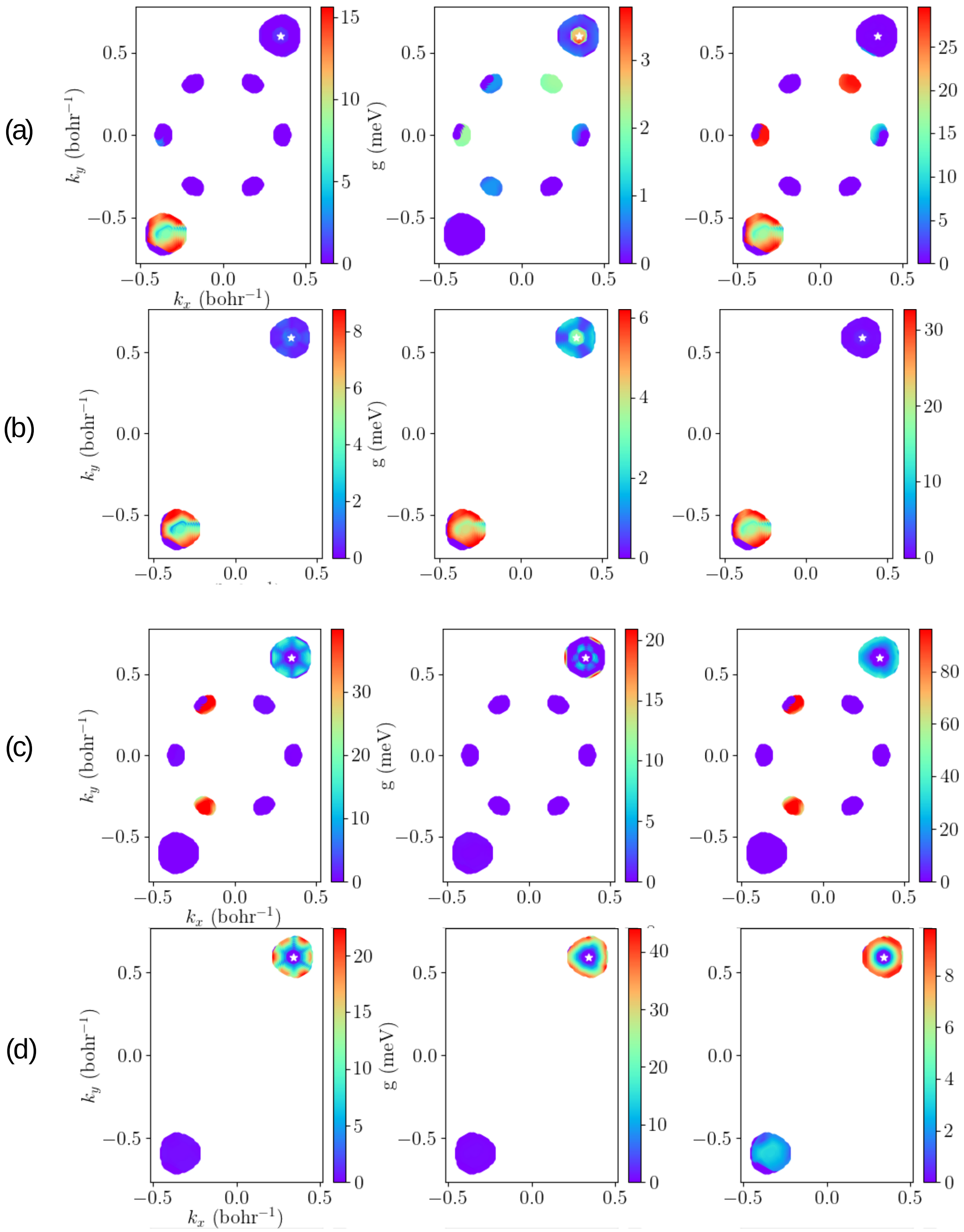

Supplementary Figure 35: Interpolated electron-phonon coupling (EPC) for three acoustic modes (i.e., LA, TA, ZA) in MoS_2. The first and third panels (a, c) refer to the unstrained material and consider different initial electronic states (white star), with one being in the lowest K (a) or K' valley (c). The second and fourth panels (b, d) show the corresponding cases for the 2% strained system, with the Q valleys absent and lying outside the thermal layer. The color plot shows the strength of the coupling to all possible final states for each phonon mode (energetically ordered from left to right).

Supplementary References

1. Smithe, K. K. H., English, C. D., Suryavanshi, S. V. & Pop, E. Intrinsic electrical transport and performance projections of synthetic monolayer MoS_2 devices. *2D Mater.* **4**, 011009 (2016).
2. Das, S., Chen, H.-Y., Penumatcha, A. V. & Appenzeller, J. High performance multilayer MoS2 transistors with scandium contacts. *Nano Lett.* **13**, 100–105 (2013).
3. Allain, A., Kang, J., Banerjee, K. & Kis, A. Electrical contacts to two-dimensional semiconductors. *Nat. Mater.* **14**, 1195–1205 (2015).
4. Cui, X. *et al.* Low-temperature ohmic contact to monolayer MoS2 by van der Waals bonded Co/h-BN electrodes. *Nano Lett.* **17**, 4781–4786 (2017).
5. Ghibaudo, G. New method for the extraction of MOSFET parameters. *Electron. Lett.* **24**, 543–545 (1988).
6. Tosun, M. *et al.* High-gain inverters based on WSe2 complementary field-effect transistors. *ACS Nano* **8**, 4948–4953 (2014).
7. Datye, I. M. *et al.* Strain-enhanced mobility of monolayer MoS_2. *Nano Lett.* **22**, 8052–8059 (2022).
8. Shin, H. *et al.* Nonconventional strain engineering for uniform biaxial tensile strain in MoS_2 thin film transistors. *ACS Nano* **18**, 4414–4423 (2024).
9. Yang, J. A. *et al.* Biaxial tensile strain enhances electron mobility of monolayer transition metal dichalcogenides. *ACS Nano* (2023).
10. Chen, Y. *et al.* Mobility enhancement of strained MoS_2 transistor on flat substrate. *ACS Nano* **17**, 14954–14962 (2023).
11. Ng, H. K. *et al.* Improving carrier mobility in two-dimensional semiconductors with rippled materials. *Nat. Electron.* **5**, 489–496 (2022).
12. Kayal, A. *et al.* Mobility enhancement in CVD-grown monolayer MoS_2 via patterned substrate-induced nonuniform straining. *Nano Lett.* **23**, 6629–6636 (2023).
13. Liu, T. *et al.* Crested two-dimensional transistors. *Nat. Nanotechnol.* **14**, 223–226 (2019).
14. Hosseini, M., Elahi, M., Pourfath, M. & Esseni, D. Strain-induced modulation of electron mobility in single-layer transition metal dichalcogenides MX_2 (M = Mo, W; X = S, Se). *IEEE Trans. Electron Devices* **62**, 3192–3198 (2015).
15. Hosseini, M., Elahi, M., Pourfath, M. & Esseni, D. Strain induced mobility modulation in single-layer MoS_2. *J. Phys. D. Appl. Phys.* **48**, (2015).
16. Sohier, T., Gibertini, M., Campi, D., Pizzi, G. & Marzari, N. Valley-engineering mobilities in two-dimensional materials. *Nano Lett.* **19**, 3723–3729 (2019).
17. Sohier, T., de Melo, P. M. M. C., Zanolli, Z. & Verstraete, M. J. The impact of valley profile on the mobility and Kerr rotation of transition metal dichalcogenides. *2D Mater.* **10**, 025006 (2023).
18. Kaasbjerg, K., Low, T. & Jauho, A.-P. Electron and hole transport in disordered monolayer MoS_2: Atomic vacancy induced short-range and Coulomb disorder scattering. *Phys. Rev. B* **100**, 115409 (2019).
19. Marzari, N., Ferretti, A. & Wolverton, C. Electronic-structure methods for materials design. *Nat. Mater.* **20**, 736–749 (2021).
